# Inflammatory Proteins in Plasma Are Associated with Severity of Alzheimer’s Disease

**DOI:** 10.1371/journal.pone.0064971

**Published:** 2013-06-10

**Authors:** Rufina Leung, Petroula Proitsi, Andrew Simmons, Katie Lunnon, Andreas Güntert, Deborah Kronenberg, Megan Pritchard, Magda Tsolaki, Patrizia Mecocci, Iwona Kloszewska, Bruno Vellas, Hilkka Soininen, Lars-Olaf Wahlund, Simon Lovestone

**Affiliations:** 1 King’s College London and National Institute for Health Research (NIHR), Biomedical Research Centres at South London and Maudsley NHS Foundation Trust and Guy’s and St. Thomas’ NHS Foundation Trust, London, United Kingdom; 2 King’s College London, Institute of Psychiatry, London, United Kingdom; 3 3rd Department of Neurology, "G.Papanicolaou" Hospital, Aristotle University of Thessaloniki, Thessaloniki, Greece; 4 Institute of Gerontology and Geriatrics, University of Perugia, Perugia, Italy; 5 Department of Old Age Psychiatry and Psychotic Disorders, Medical University of Lodz, Lodz, Poland; 6 UMR INSERM 1027, Gerontopole, CHU Toulouse, University of Toulouse, Toulouse, France; 7 University of Eastern Finland and University Hospital of Kuopio, Kuopio, Finland; 8 Department of Neurobiology, Care Sciences and Society, Section of Clinical Geriatrics, Karolinska Institutet, Karolinska University Hospital, Huddinge, Stockholm, Sweden; University of Melbourne, Australia

## Abstract

Markers of Alzheimer’s disease (AD) are being widely sought with a number of studies suggesting blood measures of inflammatory proteins as putative biomarkers. Here we report findings from a panel of 27 cytokines and related proteins in over 350 subjects with AD, subjects with Mild Cognitive Impairment (MCI) and elderly normal controls where we also have measures of longitudinal change in cognition and baseline neuroimaging measures of atrophy. In this study, we identify five inflammatory proteins associated with evidence of atrophy on MR imaging data particularly in whole brain, ventricular and entorhinal cortex measures. In addition, we observed six analytes that showed significant change (over a period of one year) in people with fast cognitive decline compared to those with intermediate and slow decline. One of these (IL-10) was also associated with brain atrophy in AD. In conclusion, IL-10 was associated with both clinical and imaging evidence of severity of disease and might therefore have potential to act as biomarker of disease progression.

## Introduction

Alzheimer’s disease (AD) is the most common form of dementia and although progress is being made, the development of disease modification therapies is currently hampered by the lack of biomarkers. There are many potential types of biomarkers, but in particular, indicators of disease progression or disease state would find utility in clinical trials, to stratify participants or to measure change over time [1]–[3]. Currently, cerebrospinal fluid (CSF) levels of tau and amyloid beta (Aβ) are the most reliable and widely used protein markers for AD. However, there are practical drawbacks of using CSF as a sample medium, in addition to poor correlation between the protein levels and disease severity [4]. Several other potential protein-based AD markers have been explored during the past decade [5], these markers have most often been analyzed in relation to diagnosis rather than disease severity or clinical progression. In addition to fluid biomarkers, neuroimaging measures, including hippocampal volume analysis, have become widely used in clinical trials [6].

Considerable evidence suggests that inflammation plays a role in the pathogenesis of AD [7], [8], and the central nervous system (CNS) contains many components of the immune system that are synthesized by astrocytes, microglia and neurons [9], [10]. Fibrillar Aβ deposition is associated with the activation of microglia [10]–[12], itself a relatively early event in the pathogenesis of AD, and the formation of the Aβ/microglia complex in early stages of AD has been reported to precede extensive tau-related neurofibrillary pathology [4], [13], [14]. The immune response of the brain is orchestrated by microglial cells which, on activation, become phagocytes and secrete a wide range of inflammatory mediators, including cytokines and chemokines, growth factors, complement molecules and adhesion molecules [15]. An increased interest in the complex network of cytokines has identified a growing number of inflammatory cytokines involved in CNS disorders, with a number of studies identifying cytokine proteins able to predict clinical AD diagnosis with high accuracy [16]–[18].

Our aim was to investigate the inflammatory response to AD in plasma samples and to examine whether plasma cytokines are associated with disease severity or disease progression. We analyzed a panel of 27 cytokines in a cohort of 351 patients with neuroimaging data available using multiplex immunoassays. The cytokine profiles of AD, MCI and control cases were compared, evaluated with respect to neuroimaging measures and to rate of decline.

## Materials and Methods

### AddNeuroMed Cohort

Samples used came from the AddNeuroMed study, a cross-European cohort for biomarker discovery. In this cohort, AD cases were assessed with a range of measures, including clinical at three monthly intervals in the first year and annually thereafter. MCI and control groups were assessed annually. The disease duration of AD cases were provided by their doctors, families and carers. All subjects were white Europeans recruited from the UK, France, Italy, Finland, Poland and Greece. The full standardized assessment in these studies includes demographic and medical information, cognitive assessment including the Mini-Mental State Examination (MMSE), Alzheimer’s Disease Assessment Scale – cognitive (ADAS-Cog), Consortium to Establish a Registry for Alzheimer’s Disease (CERAD) battery, and scales to assess function, behaviour and global levels of severity including the Clinical Dementia Rating (CDR) scale. The cohort has been previously described in [19], [20].

Informed consent was obtained from all subjects according to the Declaration of Helsinki (1991) and protocols and procedures were approved by the relevant Institutional Review Board at each collection site. All participants, or their carers where capacity was compromised and gave written consent or assent according to the laws of the relevant country. The capacity for consent was assessed by a clinician with experience in capacity assessment in the context of dementia. Exclusion criteria included other neurological or psychiatric disease, significant unstable systemic illness or organ failure and alcohol or substance misuse.

### Subjects

A total of 351 subjects were selected for this analysis grouped into 3 categories: 112 control subjects, 122 MCI patients and 117 AD patients. A second time point for biochemical analysis in relation to the progression of disease was used for this study (one year follow-up from baseline) with sample available from 104 of the 117 AD patients. The age range for subjects in the AD, MCI and control group were comparable (Table 1).

**Table 1:** Demographic and clinical parameters of AD patients, MCI and control subjects.

Variables	Controls	MCI	AD
	Baseline N = 112	Baseline N = 122	Baseline N = 117Year follow up N = 104
**Gender (F/M)**	60/52	60/62	78/39 (baseline)70/34 (year follow up)
**Age, (mean, SD)** *	72.3, (6.72)	73.9, (5.63)	76.2, (6.09)
**MMSE score (median), (±SD)** ¥	29, (1.21)	27, (1.64)	20.37, (4.68)
**CDR score (median), (±SD) ≠**	0, (0.09)	0.5, (0.05)	1, (0.48)
**ADAS-cog score (mean), (±SD)**	N/A	N/A	24.9, (9.97)
***APOε4*** ** allele presence**	N = 32	N = 35	N = 59 (baseline)N = 50 (year follow up)

Key:

MMSE = Mini-Mental State Examination; CDR = Clinical Dementia Rating; ADAS-cog = Alzheimer’s Disease Assessment Scale-cognitive subscale; Year follow up = a year follow up from baseline.

SD = Standard Deviation.

* ANOVA F = 5.72 (2,329); p = 0.0036. Scheffe test: Control v MCI p = 0.480; Control v AD p = 0.004; MCI v AD p = 0.107.

¥ ANOVA F = 221.29 (2,328); p<0.001. Scheffe test: Control v MCI p<0.001; Control v AD p<0.001; MCI v AD p<0.001.

≠ ANOVA F = 1320.56 (2,214) p<0.0001. Scheffe test: Control v MCI p<0.001; Control v AD p<0.001; MCI v AD p<0.001.

MMSE and CDR assessments were available from all 351 subjects, and ADAS-cog assessment was performed in AD patients only.

### Sample Preparation and Data Acquisition

All participants were required to fast for two hours before blood sample collection; only water or fluids containing no milk or sugar were allowed during the fasting period. Plasma samples were collected using EDTA coated tubes and centrifuged at 3000 rpm for 8 minutes at 4°C before being aliquoted and then frozen at −80°C.

Plasma samples were analyzed with a multiplex suspension array system using Bioplex Luminex 200 instrument (Bio-Rad Laboratories, Hercules, CA). The panel (Bio-Plex Human cytokine 27–Plex) consisted of the following 27 cyto- and chemokines: Interleukin (IL)-1β, IL-1ra, IL-2, IL-4, IL-5, IL-6, IL-7, IL-8, IL-9, IL-10, IL-12 (p70), IL-13,IL-15, IL-17, Eotaxin, fibroblast growth factor (FGF) basic, granulocyte-colony stimulating factor (G-CSF), granulocyte-macrophage colony stimulation factor (GM-CSF), Interferon-gamma (IFN-γ), interferon-inducible protein-10 (IP-10), monocyte chemotactic protein-1 (MCP-1), macrophage inflammatory protein (MIP)-1α, MIP-1β, platelet-derived growth factor (PDGF)-BB, Rantes, tumor necrosis factor (TNF)-α and vascular endothelial growth factor (VEGF). The overall detection concentration range according to the standard curves was 6,560–82,807 pg/ml.

The samples were prepared according to the manufacturer’s instructions. All samples and standards were run in duplicate and were measured as pg/ml. The system running protocol was set according to manufacturer’s guidelines: the protocol was set to a high RP1 (fluorescent channel) target value for CAL2 calibration and the acceptable recovery percent range to a range of 80–120%. The protocol was set to 100 beads per region and the sample volume adjusted to 50 µl. After the plate reading, the results files were generated using Bio-Plex Manager software 4 (Bio-Rad Laboratories, Hercules, CA).

### Data Processing

Some samples duplicate measures were excluded from further analysis. The exclusion was based on the observed concentration of standards that were not within the 80–120% recovery range. In order to screen outliers, we generated a correlation matrix with each subject for all 27 analytes. This gave us an output in the form of a correlation coefficient. Patients falling below an r = 0.8 correlation coefficient threshold were omitted from subsequent analysis.

### Neuroimaging

Six different 1.5 T MR systems (4 General Electric, 1 Siemens and 1 Picker) were used for data collection. Data acquisition was designed to be compatible with the Alzheimer Disease Neuroimaging Initiative (ADNI) [21]. The imaging protocol was based on using a high resolution saggital 3D T1-weighted MPRAGE volume (voxel size 1.1×1.1×1.2 mm3) and axial proton density/T2-weighted fast spin echo images. Full brain and skull coverage was required and a detailed quality control was carried out on all MR images according to the AddNeuroMed quality control procedure [20], [22]. All MR images were examined by an on-site radiologist for exclusion of any subjects with non-AD related pathologies.

### MR Image Analysis

The highly automated Freesurfer pipeline (version 4.5.0) was used to produce both regional cortical thickness measures and regional volume measures. Cortical reconstruction and volumetric segmentation included removal of non-brain tissue, automated Talairach transformation, intensity correction and segmentation of the subcortical white matter and deep gray matter volumetric structures (including hippocampus, amygdala, caudate, putamen, ventricles). Identification of the grey matter/white matter boundary was followed by surface inflation and registration to a spherical atlas which utilises individual cortical folding patterns to match cortical geometry across subjects and parcellation of the cerebral cortex into units based on gyral and sulcal structure. All regional volume measures from each subject were normalised by the subjects’ intracranial volume. Cortical thickness measures were used in their raw form.

### Statistical Analysis

Statistical analysis was performed in SPSS Version 15 (SPSS Inc., Chicago, USA) and STATA 10 (Stata Corporation, College Station, TX, USA). The Kolmogorov-Smirnov test was used to check for normal distribution of continuous outcomes. In cases of non-normality the natural log of each variable was used. Linear regression was used in order to compare baseline (visit 1) plasma cytokine levels between AD, MCI and control samples adjusting for age, gender, centre and presence of the *APOε4* allele. Multiple linear regression was also employed to investigate the relationship of neuroimaging measures, such as whole brain volume, hippocampus and entorhinal cortex with baseline cytokine levels adjusting for age, gender, centre, presence of the *APOε4* allele and disease status. Linear regression was also performed to assess the relationship of covariates (Table S1). To test whether the association of cytokines with disease status differed between subjects in different *APOε4* allele or gender strata, or whether the association of neuroimaging measures with cytokines was different amongst disease groups, we tested for interactions followed by likelihood ratio tests to compare a model assuming no interaction to a model with an interaction term. If significant interactions were identified, data was presented separately for different strata. Differences were considered significant if p≤0·05 (two-tailed).

### Association of Cognitive Decline with Changes in Cytokine Levels

AD patients were grouped into slow, intermediate and fast declining patients, based on cognitive decline slope per year in MMSE, ADAS-cog and CDR. Cognitive decline slopes were calculated using mixed linear effects models. The average baseline cognitive outcome and the average change in the cognitive outcome over follow-up time was calculated for all subjects per day as a group (fixed effects) and subject-specific intercept and slope terms which reflected deviation from the group average (mixed linear effects) were calculated. The calculation included adjustment for age at baseline, disease duration at baseline, gender, cholinesterase inhibitors, antidepressants, antipsychotics, ethnicity/centre, education, being a widow/er, being in a nursing home and presence of *APOε4* allele. Covariates significant at the p<0.10 level were included in a final model for each cognitive model. The annual cognitive decline was obtained by multiplying the slope of cognitive decline measured using days with the average number of days per year (365.25). An annual MMSE, ADAS-cog and CDR score decline of 4 or more was considered as fast decline, whereas an annual MMSE score decline of 2–4 was considered as intermediate decline; and a score below 2 was considered as slow decline.

To assess change in cytokines as a function of cognition in people with dementia, we measured cytokines in subjects with AD at year 1 as well as at baseline. Mixed linear mixed effects models as described above were used to investigate the relationship between changes in cytokine levels measured at baseline and at year 1 and cognitive decline in AD. As before, the calculation included adjustment for age at baseline, disease at baseline, gender, cholinesterease inhibitors, antidepressants, antipsychotics, ethnicity/centre, education, being a widow/er, being in a nursing home and presence of *APOε4* allele. Only age at baseline, disease duration, centre and gender were robustly associated with cytokine changes between the two visits and were therefore used as covariates. A significant interaction between the cognitive decline group and the variable indicating when cytokine levels were measured (visit) would indicate differences between the change in cytokine levels and decline group.

## Results

### Exclusions

Four analytes, namely MIP-1α, MIP-1β, RANTES (CCL5) and VEGF, were excluded from analysis as more than 50% of the subjects fell below an r = 0.8 correlation coefficient threshold suggesting technical failure. In average, across the remaining 23 analytes a range of 1–14 subjects were excluded from analysis, as they fell below the r = 0.8 correlation coefficient threshold in the correlation matrix. The number of excluded subjects was similar across the three diagnostic groups. The demographics and clinical parameters of AD patients, MCI and control subjects are presented in Table 1 and Table 2 shows the results of the 23 detectable proteins in plasma.

**Table 2:** Concentrations of analytes in plasma and association of analytes with diagnostic groups.

	ACTUAL PROTEIN LEVELS (pg/ml)								LINEAR REGRESSION (ON TRANSFORMED DATA)									LINEAR REGRESSION	
Cytokine																		(ON TRANSFORMED DATA)	
	CTL				MCI		AD		Controls-MCI			Controls-AD			MCI-AD			Whole Cohort comparison	
	Mean		Range		Mean	Range	Mean	Range	Beta	95% CI	p	Beta	95% CI	p	Beta	95% CI	p	p	
									(R^2^)			(R^2^)			(R^2^)				
**IL-1b**	3		0.99–11.97		3	1.05–9.26	3.3	1.21–13.03	0.09	−0.094	0.278	0.1	−1.755	0.257	0.01	−0.235	0.921	0.757	
									−0.004	0.235		−0.005	0.74		0	0.094			
**IL-1ra**	137.9		4.61–602.53		155.7	5.90–1698.63	221.7	6.13–5843.77	0.02	−0.285	0.894	0.01	−1.403	0.925	0	−0.26	0.972	0.451	
									0	0.26		−1.00E-04	2.882		0	0.285			
**IL-2**	10.5		0.51–50.59		11	1.52–37.93	14	0.91–192.57	0.16	−0.225	0.91	0.09	−1.272	0.51	−0.07	−0.458	0.701	0.157	
									−0.004	0.458		−0.001	3.084		−8.00E-04	0.225			
**IL-4**	3		0.65–10.52		3	0.44–12.10	2.9	0.49–8.77	−0.03	−0.155	0.679	−0.06	−1.23	0.457	−0.03	−0.175	0.717	0.469	
									−0.001	0.175		−0.002	1.409		−1.00E-04	0.155			
**IL-5**	4.8		1.04–24.57		4.4	0.83–15.17	4.3	1.00–15.82	−0.03	−0.3	0.791	−0.05	−1.93	0.651	−0.02	−0.135	0.841	0.988	
									−2.00E-04	0.135		−0.001	1.1		−1.00E-04	0.3			
**IL-6**	8.9		1.26–36.58		9.6	1.10–28.53	11.2	0.93–74.8	0.01	−0.181	0.917	0.11	−1.65	0.342	0.1	−0.278	0.382	0.472	
									0	0.278		−0.003	2.512		−0.003	0.181			
**IL-7**	6.2		1.21–23.92		6.5	1.29–28.05	6.9	0.98–37.49	0.03	−0.137	0.798	0.06	−3.376	0.584	0.03	−0.315	0.759	0.499	
									−2.00E-04	0.315		−0.001	0.699		−3.00E-04	0.137			
**IL-8**	8.3		1.92–31.34		7.5	0.93–32.89	7.5	0.63–20.14	−0.12	−0.295	0.203	−0.09	−2.181	0.362	0.03	−0.116	0.732	0.642	
									−0.005	0.116		−0.003	0.936		−3.00E-04	0.295			
**IL-9**		63.2		6.41–808.92	56.3	6.49–301.58	65.6	6.41–518.76	0.07	−0.179	0.601	0.09	−1.764	0.509	0.02	−0.345	0.872	0.244	
									−0.001	0.345		−0.002	2.394		−1.00E-04	0.179			
**IL-10**		10		0.97–37.62	9.8	0.97–42.18	12.1	1.02–49.73	0.06	−0.252	0.764	0.3	−2.14	0.134	0.25	−0.511	0.215	0.185	
									−0.001	0.511		−0.017	2.977		−0.011	0.252			
**IL-12**		15.4		1.99–128.57	14.3	1.31–41.65	18	1.24–238.34	−0.14	−0.282	0.272	−0.01	−4.147	0.933	0.13	−0.246	0.299	0.857	
									−0.004	0.246		0	−0.021		−0.004	0.281			
**IL-13**		8.9		1.74–58.41	9.9	1.37–42.13	9	1.79–41.02	0.06	−0.065	0.559	−0.05	−2.472	0.62	−0.11	−0.353	0.265	0.475	
									−0.001	0.353		−0.001	0.749		−0.004	0.065			
**IL-15**		6.7		0.88–59.14	6.4	1.18–39.57	5.9	0.79–26.69	0.2	−0.127	0.276	0.13	−3.568	0.485	−0.07	−0.591	0.688	0.065	
									−0.009	0.59		−0.004	1.172		−0.001	0.127			
**IL-17**		65.1		2.90–253.32	63.9	3.09–258.41	59.5	3.42–237.07	−0.07	−0.325	0.641	−0.17	−1.054	0.265	−0.1	−0.309	0.498	0.287	
									−0.001	0.309		−0.005	4.228		−0.001	0.325			
**Eotaxin**		79.5		11.15–291.31	77.9	7.14–318.78	87	15.25–384.91	−0.04	−0.294	0.706	0.16	−1.794	0.128	0.2	−0.131	0.045*	0.297	
									−0.001	0.131		−0.009	1.381		−0.015	0.294			
**FGF**		30.7		1.63–89.61	44.1	2.91–133.40	53.6	3.44–147.52	0.33	0.112	0.086	0.49	−0.732	0.012*	0.16	−0.893	0.387	0.298	
									−0.018	0.893		−0.038	3.641		−0.004	0.112			
**G-CSF**		73.6		16.30–143.37	74.4	11.35–187.81	73.7	19.31–168.61	−0.05	−0.137	0.501	−0.09	−1.554	0.231	−0.04	−0.147	0.576	0.404	
									−0.002	0.147		−0.005	0.656		−0.001	0.137			
**GM-CSF**		47.1		0.60–164.87	47.1	2.36–181.64	47.7	2.13–291.80	0.06	−0.23	0.674	−0.04	−5.12	0.797	−0.1	−0.312	0.485	0.598	
									−0.001	0.312		2.00E-04	−0.208		0.002	0.23			
**IFN-γ**		170.6		13.18–1032.07	171	3.42–791.40	202.2	7.94–1884.05	0.03	−0.214	0.825	−0.06	−3.04	0.654	−0.09	−0.308	0.488	0.625	
									−1.00E-04	0.308		−0.001	1.8		−0.001	0.214			
**IP-10**		605.9		131.77–2105.65	567	25.40–2448.32	629.6	149.75–2203.04	−0.05	−0.208	0.535	−0.01	−1.81	0.924	0.04	−0.099	0.593	0.56	
									−0.001	0.1		0	0.991		−0.001	0.208			
**MCP-1**		32.5		5.70–99.22	30.3	6.78–65.61	34.8	2.51–105.24	0.06	−0.166	0.465	0.14	−2.555	0.109	0.08	−0.187	0.365	0.72	
									−0.002	0.187		−0.009	0.601		−0.003	0.166			
**PDGF**		3205.2		49.84–15964.6	3353.5	33.96–15300.08	2989.9	54.40–10105.77	−0.17	−0.324	0.238	−0.25	−3.79	0.099	0.08	−0.262	0.593	0.445	
									−0.004	0.262		−0.008	0.8		−8.00E-04	0.324			
**TNF-α**		81.6		5.44–374.58	74.4	8.31–276.37	79.5	6.65–328.53	−0.12	−0.304	0.279	−0.09	−0.811	0.399	0.02	−0.151	0.828	0.535	
									−0.004	0.151		−0.002	2.63		−2.00E-04	0.304			

Whole cohort presents results for the whole cohort and was based on all three diagnostic groups. Plasma protein levels were generally higher in AD than in MCI or control with a few exceptions. The relationship between cytokines and diagnostic groups was assessed by linear regression adjusting for age, gender, collection site and presence of the *APOε4* allelle. In this analysis beta values are for the log transformed data. Key: Beta = regression coefficient (slope) on log- transformed data; 95% CI = 95% confidence interval; R^2^ = R^2^ value (coefficient of determination) after adjusting for covariates; *p<0.05.

### Peripheral Cytokine Levels Reflect the Inflammatory Process Associated with AD

First, in order to evaluate whether a different extent of inflammation in AD, MCI and controls is reflected in the respective plasma samples, baseline cytokine levels between the different groups were compared. The level of FGF (p = 0.012) was significantly different between AD and control subjects; whereas Eotaxin (p = 0.045) was significantly different between AD and MCI (Table 2).

### Inflammatory Proteins in Plasma are Associated with Pathological Severity

Regression analyses were performed to examine the relationship between inflammatory cytokines and disease severity as measured by atrophy on MRI measures. We first assessed the relationship between multiple cytokine levels and neuroimaging measures of whole brain volume, entorhinal cortex, entorhinal cortex thickness, hippocampal volume and ventricular volume in the whole data set sample. There was no significant relationship between multiple cytokine levels and neuroimaging measures except for TNF-α (p = 0.045), which correlated with left hippocampal volume (data not shown).

**Figure 1:**
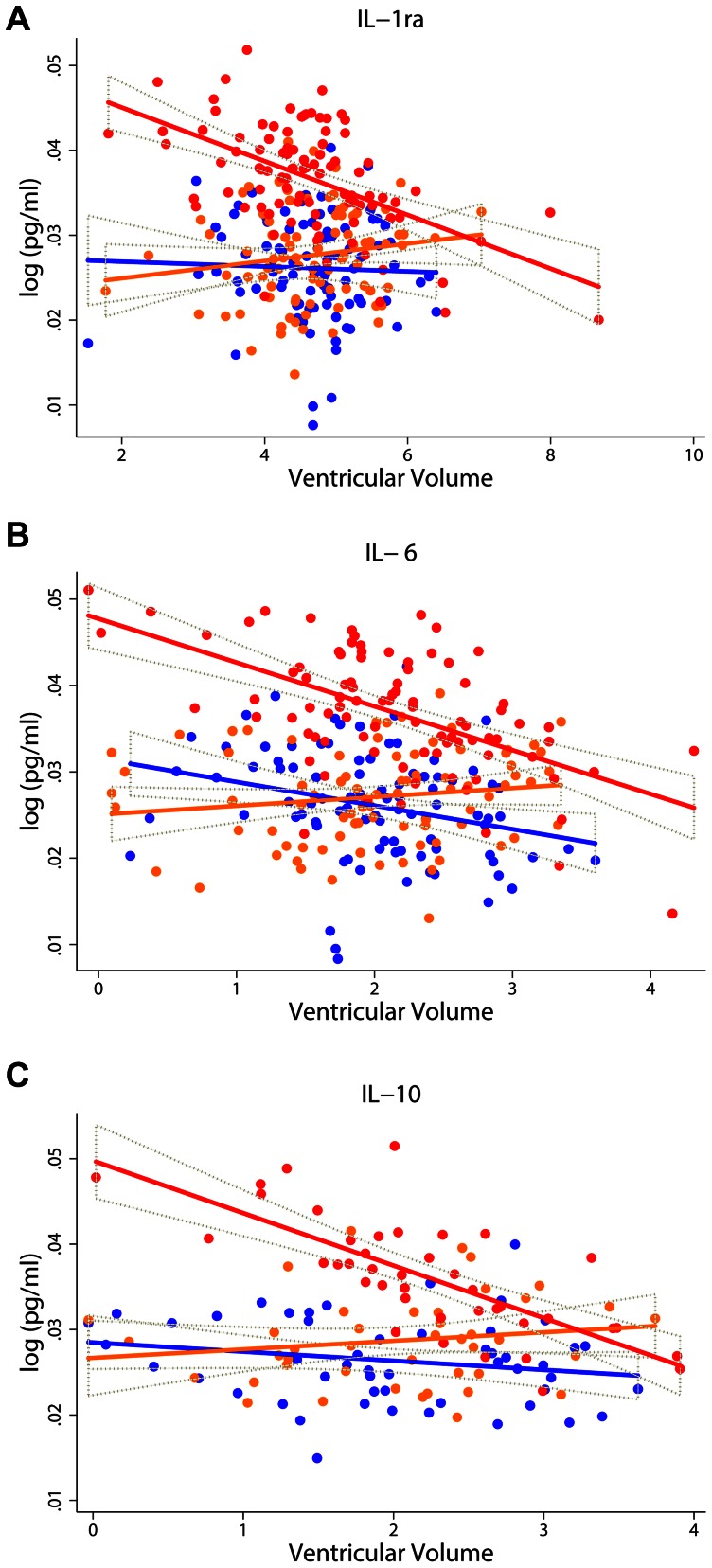
Association between inflammatory proteins levels measured in plasma versus ventricular volume. Scatter diagram with regression lines show the relationship between proteins levels of IL-1ra (N = 86) (figure 1A), IL-6 (N = 83) (figure 1B) and IL-10 (N = 38)(figure 1C)and ventricular volume in the three separate diagnostic groups. Keys: Blue line and dots denote control samples; orange line and dots denote MCI samples; red line and dots denote AD samples; brown dotted line denotes 95% CI.

We then examined whether association of cytokines with MRI measurements differed between the different diagnostic groups. Significant interactions in the ventricular volume, whole brain volume and entorhinal cortex were observed between four cytokines [IL-6 (p = 0.005), IL-10 (p = 0.025), IL-13 (p = 0.028) and IL-1ra (p = 0.002)] and clinical status (Table S2). Although we did not observe any associations between these cytokines and neuroimaging measurements for the whole cohort, significant associations between these five cytokines and neuroimaging measures were observed in AD patients (Table 3, 4). Specifically, three measures (IL-1ra, IL-6 and IL-10) significantly associated with ventricular volume in AD (Figure 1).

**Table 3:** Summary statistics for the multiple linear regression model assessing the relationship between inflammatory proteins and ventricular volume.

Cytokine	Whole cohort			Controls			MCI			AD		
(pg/ml)	Beta	95% CI	p value	Beta (R^2^)	95% CI	p value	Beta (R^2^)	95% CI	p value	Beta (R^2^)	95% CI	p value
	(R^2^)											
IL-1ra	2.00E-04	−0.002	0.77	0.003	−0.001	0.099	0.002	−0.001	0.223	−0.003	−0.006	0.021*
	(−0.008)	0.001		−0.032	0.006		−0.013	0.004		−0.068	−8.80E-04	
IL-6	−0.001	−0.003	0.151	−2.10E-03	−0.004	0.904	0.001	−0.002	0.405	−0.005	−0.009	0.018*
	−0.033	0.001		−0.028	0.004		−0.016	0.004		−0.101	−0.001	
IL-10	−0.001	−0.003	0.431	−1.10E-03	−0.004	0.809	0.001	−0.004	0.805	−0.006	−0.011	0.028*
	(−0.031)	0.001		−0.031	0.003		−0.059	0.005		−0.103	−0.001	

Whole cohort presents results for the whole cohort and was based on all three diagnostic groups with 255 subjects for IL-1ra, 250 subjects for IL-6 and 128 subjects for IL-10.

The relationship between cytokines and MRI measures was assessed by multiple linear regression adjusting for age, gender, collection site and presence of the *APOε4* allelle.

Key: Beta = regression coefficient (slope) on log- transformed data; 95% CI = 95% confidence interval; R^2^ = R^2^ value (coefficient of determination) after adjusting for covariates;

* p<0.05; Ventricular Volume is normalized to Intracranial Volume (ICV).

**Table 4:** Summary statistics for the multiple linear regression model assessing the relationship between inflammatory proteins and brain MRI measures (whole brain volume and left entorhinal cortex).

Cytokine	Whole cohort			Controls			MCI			AD		
(pg/ml)	Beta	95% CI	p value	Beta (R^2^)	95% CI	p value	Beta	95% CI	p value	Beta	95% CI	p value
	(R^2^)						(R^2^)			(R^2^)		
^W^TNF-α	−0.005	−0.011	0.081	−0.001	−0.01	0.754	−0.003	−0.014	0.613	−0.01	−0.02	0.047*
	−0.008	0.001		(−0.042)	0.007		−0.002	0.008		−0.04	7.80E-03	
^E^IL-13	−1.00E-04	−6.50E-05	0.171	4.10E-04	−4.80E-05	0.389	−1.20E-05	9.10E-05	0.351	−9.00E-05	−1.80E-04	0.048*
	−0.005	3.30E-05		−0.008	1.30E-04		−0.01	6.70E-05		−0.048	4.70E-06	

The relationship between cytokines and MRI measures was assessed by multiple linear regression adjusting for age, gender, collection site and presence of the *APOε4* allelle. Whole cohort result shows the model overall data and was based on all three diagnostic groups with 239 subjects for TNF-α and 241 subjects for IL-13.

Key: Beta = regression coefficient (slope) on transformed data; CI = 95% confidence interval; R^2^ = R^2^ value (coefficient of determination) after adjusting for covariates;

* p<0.05; ^W^ indicates whole brain volume (normalized to Intracranial Volume (ICV)); ^E^ indicates left entorhinal cortex(normalized to Intracranial Volume (ICV)).

### Inflammatory Proteins in Plasma are Associated with Rate of Cognitive Decline in AD

**Figure 2:**
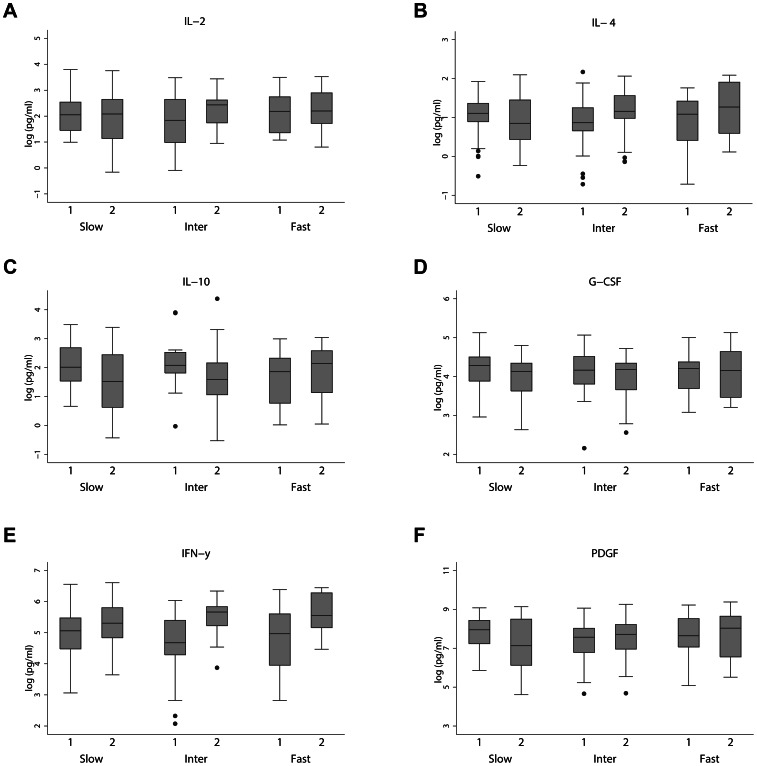
Inflammatory proteins levels at Visit 1 (baseline) and Visit 2 (one year follow up from baseline) for the three ADAS-cog cognitive decline groups. Linear mixed effects models indicated significant yearly changes in the log-transformed IL-2 (figure 2A), IL-4 (figure 2B), IL-10 (figure 2C), G-CSF (figure 2D), IFN-γ (figure 2E), and PDGF (figure 2F) (pg/ml) inflammatory protein levels between the three cognitive decline groups, after adjusting for covariates. Slow indicates slow decliner. Inter indicates intermediate decliner. Fast indicates fast decliner Key: 1– Visit 1(Baseline); 2– Visit 2 (one year follow up from baseline).

We then compared changes in the inflammatory signals between baseline and year 1 visit in participants with different rates of clinical decline, as measured with the MMSE, ADAS-cog and CDR. Overall, we observed significant changes in levels of a number of cytokines between patients with a fast or intermediate cognitive decline and patients with slow cognitive decline, particularly measured with the ADAS-cog. Specifically, we found a significant increase in the levels of IL-4 (p = 0.024), IL-10 (p = 0.040) and G-CSF (p = 0.046) in AD patients with a fast cognitive decline compared to slow cognitive decline in ADAS-cog over one year (Figure 2 and Table 5). AD patients with intermediate cognitive decline showed significantly higher levels of IL-2 (p = 0.021), IL-4 (p = 0.016), IFN-γ (p = 0.018) and PDGF (p = 0.031) compared to those with slow cognitive decline over one year (Figure 2 and Table 5).

**Table 5:** Summary of longitudinal changes in inflammatory protein levels between cognitive decline groups as measured by ADAS-cog.

	ACTUAL PROTEIN LEVELS (pg/ml)						LINER MIXED EFFECTS MODEL					
Cytokine	Slow		Intermediate		Fast		Slow versus Inter^¥^			Slow versus fast^¥^		
	ADAS-Cog Decliners		ADAS-Cog Decliners		ADAS-Cog Decliners							
	Mean (SD) [N]		Mean (SD) [N]		Mean (SD) [N]							
(pg/ml)	Baseline	Year 1	Baseline	Year 1	Baseline	Year 1	Beta	95% CI	p value	Beta	95% CI	p value
**IL-2**	11.73	9.64	9.82	11.86	19.45	12.17		0.105	0.021*		−0.511	
	−13.27	−8.09	−9.09	−7.74	−39.57	−69.55	0.691	1.278		0.086	0.683	0.777
	[77]	[83]	[44]	[47]	[23]	[22]						
**IL-4**	3.11	3.02	2.78	3.54	2.9	3.96		0.074	0.016*		0.051	
	(1.31)	−1.77	−1.73	−1.72	−1.55	−2.33	0.392	0.713		0.387	0.729	0.024*
	[114]	[91]	[64]	[51]	[31]	[24]						
**IL-10**	11.38	7.69	14.17	10.45	7.59	9.39		−0.654	0.724		0.037	
	−8.81	−7.65	−145.96	−18.1	−6.24	−6.2	0.143	0.941		0.825	1.627	0.040*
	[54]	[67]	[29]	[37]	[16]	[18]						
**G-CSF**	76.76	58.79	72.21	61.81	65.4	68.8		−0.036	0.084		0.006	
	−38.1	−28.14	−36.83	−26.26	−30.52	−39.29	0.265	0.564		0.316	0.627	0.046*
	[112]	[89]	[63]	[49]	[31]	[24]						
**IFN-γ**	209.12	241.6	178.56	270.62	235.41	319.21		0.086	0.018*		−0.069	
	−241.6	−146.43	−222.89	−132.29	(334.19)	−194.33	0.493	0.908		0.347	0.774	0.102
	[116]	[91]	[65]	[51]	[33]	[24]						
**PDGF**	3135.09	2536.81	2422.27	2823.29	3149.69	3628.44		0.065	0.031*		−0.057	
	−1969.2	−2481.5	−2079.5	−2769.5	−2760.1	−3668.9	0.731	1.399		0.62	1.3	0.073
	[111]	[85]	[64]	[46]	[32]	[23]						

The relationship between change in cytokine levels over one year and AD cognitive decline group was assessed by mixed effect model adjusting for age, gender, *APOε4* allelle, collection site and disease duration. Slow indicates slow decliner. Inter indicates intermediate decliner. Fast indicates fast decliner. Key: Beta = regression coefficient (slope) on transformed data; 95%CI = 95% confidence interval;N = number of sample; ¥ - coefficients represent the difference in the slopes for the given cytokine between cognitive decline groups across the two time points (i.e. interaction between time and the rate of decline groups).

* p<0.05.

## Discussion

We investigated plasma levels of cytokines in AD, MCI and control samples to determine whether inflammatory proteins are associated with disease progression or disease severity as assessed by memory test scores or by neuroimaging data respectively. AD is characterized by early memory loss with the first sites of pathological change measurable in life being the hippocampus and the entorhinal cortex [23], [24]. Around 80–90% of AD subjects show atrophy in both sites compared to only 5–10% of control subjects [25], [26]. Our study identified a number of analytes that correlate with brain imaging data - five analytes being associated with ventricular, whole brain and entorhinal measures of atrophy. In addition, six analytes were associated with rates of decline in cognitive scores.

A number of studies have reported that Aβ deposition can activate microglia and induce the production of IL-1, IL-6, TNF-α and MCP-1 in the AD brain [7], [27]–[32]. Aβ-induced secretion of IFN-γ and IL-1β has also been observed [33]. Grammas & Ovase demonstrated a high level of production of IL-1β, IL-6, MCP-1 and TNF-α in AD brain microvessels compared to control [34]. McGeer & Zhao showed IL-10 can be produced by microglia [35], [36]. Moreover, Soares’s study observed an elevation of IL-13 level was associated with *APOε4* allele; a known risk factor for AD [37]. The elevation of these inflammatory proteins in AD brain microvessels suggest that these proteins may play a role in neuronal damage.

Our study finds IL-6, TNF-α and three addition analytes (IL-1ra, IL-10 and IL-13) in plasma significantly inversely correlated with ventricular volume, whole brain volume or entorhinal cortex in AD. Furthermore, we observe an increase in the level of IL-10 between visit 1 and visit 2 in ADAS-cog fast decliners compare to slow decliners and, an increase level of IFN-γ was observed between visit 1 and visit 2 in ADAS-cog intermediate decliners compared to slow decliners.

Plasma cytokines are known to communicate with the brain [38] and circulating levels of peripheral cytokines have been shown to correlate and reflect central cytokine levels in the brain [38], [39]. An important but unanswered question regards the source of the inflammatory signature in the periphery in AD – is it independent of, or secondary to, the inflammatory reaction in the brain? There are various routes whereby an inflammatory activity may communicate between brain and the periphery [38]. One of these routes involves diffusion of cytokines between blood and brain in regions with an impaired blood brain barrier (BBB). In some cases cytokines can be actively transported across the BBB [39]. Another route involves cytokine activation of the endothelium signalling to macrophages in brain [40]. Understanding which, if any of these mechanisms underlies the peripheral signature of inflammatory proteins in AD is an important topic for further investigation.

The current, and these earlier studies show clearly that there is an inflammatory change in the brain in AD and an inflammatory signal in the periphery although how these are connected and the exact nature of the inflammatory profile are both far from certain. However, although it is clear from this and from other studies that there is a peripheral inflammatory response in AD, the question remains as to whether it might be sufficiently robust to act as a biomarker. One study reported 18 plasma proteins that could predict clinical AD diagnosis with high accuracy [16]. A bioinformatics-based follow-up showed that reducing the panel to 5 proteins improved accuracy to 96% [41]. However, this finding has not been widely replicated. Individual cytokines found by Ray *et al*, are also reported in other studies – for example, IL-6 and TNF-α associated with cognitive decline [42]. Our multiplex assay included only two (TNF-α and G-CSF) of the 18 proteins described by Ray *et al* but we do find G-CSF to be associated with decline on cognitive tests and to be significantly different between slow and fast declining AD patients. G-CSF plays an interesting role in the inflammatory response in AD as it suppresses the production or activity of proinflammatory cytokines [43]. A recent investigation showed decreased plasma G-CSF levels in early AD [44], in contrast to the finding of Ray *et al*. In the current study we find a significantly higher expression of G-CSF in fast compared to slow declining AD patients.

One limitation of the current study is that no adjustment of multiple testing was performed. However, given the modest effect sizes observed, correction for multiple testing whilst performing such large number of analyses beyond looking for associations between cytokines and AD status (such as rate of decline, measures of disease in the brain and change in time of the analytes in relation to disease progression) would require study sizes considerably larger than ours, and probably any existing biomarker cohort currently existing. We draw from this two conclusions - first that the data presented here should be considered preliminary and requiring replication and secondly, that as in genomic studies, cohorts for biomarker analyses will have to increase substantially. Nonetheless, this study is one of the first to examine large numbers of functionally related analytes in a large cohort of subjects in relation to multiple measures of disease severity. Moreover, our finding of an association between an inflammatory profile including some markers previously associated with AD, suggests that the inflammatory change in the periphery does occur in AD and is worthy of further investigation and in particular that IL-2, IL-4, IL-10, G-CSF and IFN-γ may be markers not of disease per se but of disease severity.

## Supporting Information

Table S1 **Association between inflammatory proteins and covariates.** Most proteins showed a significant association with the covariate of collection site, with the exception of IL9, IL10, IL15 and IP-10. The number of proteins showing a significant association with the covariates of age and gender was very low (N = 2 and N = 1 respectively). *p<0.05; **p<0.001.
(DOC)

Table S2 **Summary of significant interaction between inflammatory proteins and diagnostic groups**. The relationship between cytokines and MRI measures in diagnostic groups was assessed by linear regression adjusting for age, gender, collection site and presence of the *APOε4* allelle. *p<0.05; **p<0.01.
(DOC)

## References

[1] Bailey P (2007) Biological markers in Alzheimer’s disease. Can J Neurol Sci 34 Suppl 1S72–76. 17469687 10.1017/s0317167100005618

[2] Lovestone S, Guntert A, Hye A, Lynham S, Thambisetty M, et al (2007) Proteomics of Alzheimer’s disease: understanding mechanisms and seeking biomarkers. Expert Rev Proteomics 4: 227–238. 17425458 10.1586/14789450.4.2.227

[3] Noelker C, Hampel H, Dodel R (2011) Blood-based protein biomarkers for diagnosis and classification of neurodegenerative diseases: current progress and clinical potential. Mol Diagn Ther 15: 83–102. 21623645 10.1007/BF03256398

[4] Arends YM, Duyckaerts C, Rozemuller JM, Eikelenboom P, Hauw JJ (2000) Microglia, amyloid and dementia in alzheimer disease. A correlative study. Neurobiol Aging 21: 39–47. 10794847 10.1016/s0197-4580(00)00094-4

[5] Shaw LM, Korecka M, Clark CM, Lee VM, Trojanowski JQ (2007) Biomarkers of neurodegeneration for diagnosis and monitoring therapeutics. Nat Rev Drug Discov 6: 295–303. 17347655 10.1038/nrd2176

[6] Petrella JR, Coleman RE, Doraiswamy PM (2003) Neuroimaging and early diagnosis of Alzheimer disease: a look to the future. Radiology 226: 315–336. 12563122 10.1148/radiol.2262011600

[7] Akiyama H, Barger S, Barnum S, Bradt B, Bauer J, et al. ( (2000) Inflammation and Alzheimer’s disease. Neurobiol Aging 21: 383–421. 10858586 10.1016/s0197-4580(00)00124-x PMC3887148

[8] Mrak RE, Griffin WS (2005) Potential inflammatory biomarkers in Alzheimer’s disease. J Alzheimers Dis 8: 369–375. 16556968 10.3233/jad-2005-8406

[9] Bonifati DM, Kishore U (2007) Role of complement in neurodegeneration and neuroinflammation. Mol Immunol 44: 999–1010. 16698083 10.1016/j.molimm.2006.03.007

[10] Eikelenboom P, Veerhuis R (1996) The role of complement and activated microglia in the pathogenesis of Alzheimer’s disease. Neurobiol Aging 17: 673–680. 8892339 10.1016/0197-4580(96)00108-x

[11] Itagaki S, McGeer PL, Akiyama H, Zhu S, Selkoe D (1989) Relationship of microglia and astrocytes to amyloid deposits of Alzheimer disease. J Neuroimmunol 24: 173–182. 2808689 10.1016/0165-5728(89)90115-x

[12] Rozemuller JM, Eikelenboom P, Stam FC, Beyreuther K, Masters CL (1989) A4 protein in Alzheimer’s disease: primary and secondary cellular events in extracellular amyloid deposition. J Neuropathol Exp Neurol 48: 674–691. 2677252 10.1097/00005072-198911000-00009

[13] Eikelenboom P, Veerhuis R, Scheper W, Rozemuller AJ, van Gool WA, et al (2006) The significance of neuroinflammation in understanding Alzheimer’s disease. J Neural Transm 113: 1685–1695. 17036175 10.1007/s00702-006-0575-6

[14] Vehmas AK, Kawas CH, Stewart WF, Troncoso JC (2003) Immune reactive cells in senile plaques and cognitive decline in Alzheimer’s disease. Neurobiol Aging 24: 321–331. 12498966 10.1016/s0197-4580(02)00090-8

[15] Griffin WS (2006) Inflammation and neurodegenerative diseases. Am J Clin Nutr 83: 470S–474S. 16470015 10.1093/ajcn/83.2.470S

[16] Ray S, Britschgi M, Herbert C, Takeda-Uchimura Y, Boxer A, et al (2007) Classification and prediction of clinical Alzheimer’s diagnosis based on plasma signaling proteins. Nat Med 13: 1359–1362. 17934472 10.1038/nm1653

[17] Soares HD, Potter WZ, Pickering E, Kuhn M, Immermann FW, et al (2012) Plasma biomarkers associated with the apolipoprotein E genotype and Alzheimer disease. Arch Neurol 69: 1310–1317. 22801723 10.1001/archneurol.2012.1070 PMC3683865

[18] Doecke JD, Laws SM, Faux NG, Wilson W, Burnham SC, et al (2012) Blood-based protein biomarkers for diagnosis of Alzheimer disease. Arch Neurol 69: 1318–1325. 22801742 10.1001/archneurol.2012.1282 PMC6287606

[19] Lovestone S, Francis P, Kloszewska I, Mecocci P, Simmons A, et al (2009) AddNeuroMed–the European collaboration for the discovery of novel biomarkers for Alzheimer’s disease. Ann N Y Acad Sci 1180: 36–46. 19906259 10.1111/j.1749-6632.2009.05064.x

[20] Simmons A, Westman E, Muehlboeck S, Mecocci P, Vellas B, et al (2009) MRI measures of Alzheimer’s disease and the AddNeuroMed study. Ann N Y Acad Sci 1180: 47–55. 19906260 10.1111/j.1749-6632.2009.05063.x

[21] Jack CR Jr, Bernstein MA, Fox NC, Thompson P, Alexander G, et al (2008) The Alzheimer’s Disease Neuroimaging Initiative (ADNI): MRI methods. J Magn Reson Imaging 27: 685–691. 18302232 10.1002/jmri.21049 PMC2544629

[22] Simmons A, Westman E, Muehlboeck S, Mecocci P, Vellas B, et al (2011) The AddNeuroMed framework for multi-centre MRI assessment of Alzheimer’s disease: experience from the first 24 months. Int J Geriatr Psychiatry 26: 75–82. 21157852 10.1002/gps.2491

[23] Jobst KA, Smith AD, Szatmari M, Molyneux A, Esiri ME, et al (1992) Detection in life of confirmed Alzheimer’s disease using a simple measurement of medial temporal lobe atrophy by computed tomography. Lancet 340: 1179–1183. 1359259 10.1016/0140-6736(92)92890-r

[24] O’Brien JT (2007) Role of imaging techniques in the diagnosis of dementia. Br J Radiol 80 Spec No 2: S71–77. 10.1259/bjr/33117326 18445747

[25] Barber R, Gholkar A, Scheltens P, Ballard C, McKeith IG, et al (1999) Medial temporal lobe atrophy on MRI in dementia with Lewy bodies. Neurology 52: 1153–1158. 10214736 10.1212/wnl.52.6.1153

[26] Scheltens P, Fox N, Barkhof F, De Carli C (2002) Structural magnetic resonance imaging in the practical assessment of dementia: beyond exclusion. Lancet Neurol 1: 13–21. 12849541 10.1016/s1474-4422(02)00002-9

[27] Frei K, Malipiero UV, Leist TP, Zinkernagel RM, Schwab ME, et al (1989) On the cellular source and function of interleukin 6 produced in the central nervous system in viral diseases. Eur J Immunol 19: 689–694. 2543584 10.1002/eji.1830190418

[28] Haga S, Ikeda K, Sato M, Ishii T (1993) Synthetic Alzheimer amyloid beta/A4 peptides enhance production of complement C3 component by cultured microglial cells. Brain Res 601: 88–94. 8431789 10.1016/0006-8993(93)91698-r

[29] Ishizuka K, Kimura T, Igata-yi R, Katsuragi S, Takamatsu J, et al (1997) Identification of monocyte chemoattractant protein-1 in senile plaques and reactive microglia of Alzheimer’s disease. Psychiatry Clin Neurosci 51: 135–138. 9225377 10.1111/j.1440-1819.1997.tb02375.x

[30] McGeer PL, Schulzer M, McGeer EG (1996) Arthritis and anti-inflammatory agents as possible protective factors for Alzheimer’s disease: a review of 17 epidemiologic studies. Neurology 47: 425–432. 8757015 10.1212/wnl.47.2.425

[31] Meda L, Baron P, Prat E, Scarpini E, Scarlato G, et al (1999) Proinflammatory profile of cytokine production by human monocytes and murine microglia stimulated with beta-amyloid[25–35]. J Neuroimmunol 93: 45–52. 10378868 10.1016/s0165-5728(98)00188-x

[32] Yao J, Keri JE, Taffs RE, Colton CA (1992) Characterization of interleukin-1 production by microglia in culture. Brain Res 591: 88–93. 1446236 10.1016/0006-8993(92)90981-e

[33] Suo Z, Tan J, Placzek A, Crawford F, Fang C, et al (1998) Alzheimer’s beta-amyloid peptides induce inflammatory cascade in human vascular cells: the roles of cytokines and CD40. Brain Res 807: 110–117. 9757011 10.1016/s0006-8993(98)00780-x

[34] Grammas P, Ovase R (2001) Inflammatory factors are elevated in brain microvessels in Alzheimer’s disease. Neurobiol Aging 22: 837–842. 11754990 10.1016/s0197-4580(01)00276-7

[35] McGeer PL, McGeer EG (1995) The inflammatory response system of brain: implications for therapy of Alzheimer and other neurodegenerative diseases. Brain Res Brain Res Rev 21: 195–218. 8866675 10.1016/0165-0173(95)00011-9

[36] Zhao B, Schwartz JP (1998) Involvement of cytokines in normal CNS development and neurological diseases: recent progress and perspectives. J Neurosci Res 52: 7–16. 9556025 10.1002/(SICI)1097-4547(19980401)52:1<7::AID-JNR2>3.0.CO;2-I

[37] Soares HD, Potter WZ, Pickering E, Kuhn M, Immermann FW, et al.. (2012) Plasma Biomarkers Associated With the Apolipoprotein E Genotype and Alzheimer Disease. Arch Neurol: 1–8. 10.1001/archneurol.2012.1070 PMC3683865 22801723

[38] Konsman JP, Parnet P, Dantzer R (2002) Cytokine-induced sickness behaviour: mechanisms and implications. Trends Neurosci 25: 154–159. 11852148 10.1016/s0166-2236(00)02088-9

[39] Banks WA, Farr SA, Morley JE (2002) Entry of blood-borne cytokines into the central nervous system: effects on cognitive processes. Neuroimmunomodulation 10: 319–327. 12907838 10.1159/000071472

[40] Perry VH (2004) The influence of systemic inflammation on inflammation in the brain: implications for chronic neurodegenerative disease. Brain Behav Immun 18: 407–413. 15265532 10.1016/j.bbi.2004.01.004

[41] Gomez Ravetti M, Moscato P (2008) Identification of a 5-protein biomarker molecular signature for predicting Alzheimer’s disease. PLoS One 3: e3111. 18769539 10.1371/journal.pone.0003111 PMC2518833

[42] Dziedzic T (2006) Systemic inflammatory markers and risk of dementia. Am J Alzheimers Dis Other Demen 21: 258–262. 16948290 10.1177/1533317506289260 PMC10833275

[43] Sanchez-Ramos J, Song S, Sava V, Catlow B, Lin X, et al (2009) Granulocyte colony stimulating factor decreases brain amyloid burden and reverses cognitive impairment in Alzheimer’s mice. Neuroscience 163: 55–72. 19500657 10.1016/j.neuroscience.2009.05.071 PMC5966834

[44] Laske C, Stellos K, Stransky E, Leyhe T, Gawaz M (2009) Decreased plasma levels of granulocyte-colony stimulating factor (G-CSF) in patients with early Alzheimer’s disease. J Alzheimers Dis 17: 115–123. 19494436 10.3233/JAD-2009-1017

