# Supplementary material for: Inflammatory Proteins in Plasma Are Associated with Severity of Alzheimer’s Disease
Source: PLoS One. 2013 Jun 10;8(6):e64971. doi: 10.1371/journal.pone.0064971 (PMC3677891; doi:10.1371/journal.pone.0064971)
Supplement: Table S1 — Association between inflammatory proteins and covariates. Most proteins showed a significant association with the covariate of collection site, with the exception of IL9, IL10, IL15 and IP-10. The number of proteins showing a significant association with the covariates of age and gender was very low (N = 2 and N = 1 respectively). *p<0.05; **p<0.001. (DOC) [file pone.0064971.s001.doc]

| **Cytokine** | **N** | **Age** | **Gender** | ***APOε4* carriers** | **Centre** |
| --- | --- | --- | --- | --- | --- |
| **(pg/ml)** |  | **p value** | **p value** | **p value** | **p value** |
| **IL-1b** | 268 | 0.029* | 0.051 | 0.411 | <0.001** |
| **IL-1ra** | 305 | 0.861 | 0.473 | 0.041* | <0.001** |
| **IL-2** | 214 | 0.422 | 0.299 | 0.973 | 0.032* |
| **IL-4** | 315 | 0.239 | 0.13 | 0.010* | <0.001** |
| **IL-5** | 273 | 0.183 | 0.402 | 0.036* | <0.001** |
| **IL-6** | 299 | 0.298 | 0.224 | 0.113 | <0.001** |
| **IL-7** | 296 | 0.246 | 0.611 | 0.003* | <0.001** |
| **IL-8** | 283 | 0.051 | 0.069 | 0.015* | <0.001** |
| **IL-9** | 303 | 0.496 | 0.956 | 0.34 | 0.132 |
| **IL-10** | 157 | 0.432 | 0.605 | 0.27 | 0.508 |
| **IL-12** | 297 | 0.043* | 0.197 | 0.023* | <0.001** |
| **IL-13** | 287 | 0.733 | 0.079 | 0.005* | <0.001** |
| **IL-15** | 154 | 0.524 | 0.203 | 0.86 | 0.226 |
| **IL-17** | 251 | 0.125 | 0.514 | 0.001** | <0.001** |
| **Eotaxin** | 249 | 0.141 | 0.008* | 0.653 | 0.001** |
| **FGF** | 173 | 0.733 | 0.473 | 0.764 | 0.008* |
| **G-CSF** | 304 | 0.829 | 0.74 | 0.091 | <0.001** |
| **GM-CSF** | 298 | 0.47 | 0.578 | 0.046* | 0.001** |
| **IFN-γ** | 314 | 0.466 | 0.046* | 0.277 | <0.001** |
| **IP-10** | 305 | 0.006* | 0.825 | 0.453 | 0.104 |
| **MCP-1** | 301 | 0.799 | 0.912 | 0.086 | 0.022* |
| **PDGF** | 305 | 0.48 | 0.16 | 0.018* | <0.001** |
| **TNF-α** | 281 | 0.565 | 0.673 | 0.001** | <0.001** |
