# Supplementary material for: Inflammatory Proteins in Plasma Are Associated with Severity of Alzheimer’s Disease
Source: PLoS One. 2013 Jun 10;8(6):e64971. doi: 10.1371/journal.pone.0064971 (PMC3677891; doi:10.1371/journal.pone.0064971)
Supplement: Table S2 — Summary of significant interaction between inflammatory proteins and diagnostic groups. The relationship between cytokines and MRI measures in diagnostic groups was assessed by linear regression adjusting for age, gender, collection site and presence of the APOε4 allelle. *p<0.05; **p<0.01. (DOC) [file pone.0064971.s002.doc]

| **Cytokine (pg/ml)** | **Ventricular volume** | | | **Whole Brain volume** | | | **Left Entorhinal Cortex** | | |
| --- | --- | --- | --- | --- | --- | --- | --- | --- | --- |
|  | **MCI-CTL** | **AD-CTL** | **AD-MCI** | **MCI-CTL** | **AD-CTL** | **AD-MCI** | **MCI-CTL** | **AD-CTL** | **AD-MCI** |
|  | **p value** | **p value** | **p value** | **p value** | **p value** | **p value** | **p value** | **p value** | **p value** |
| **IL-1ra** | 0.62 | 0.002** | 0.007** | 0.316 | 0.049* | 0.356 | 0.285 | 0.477 | 0.634 |
| N=86 |  |  |  |  |  |  |  |  |  |
| **IL-6** | 0.448 | 0.063 | 0.005** | 0.873 | 0.764 | 0.879 | 0.553 | 0.108 | 0.371 |
| N=83 |  |  |  |  |  |  |  |  |  |
| **IL-10** | 0.365 | 0.098 | 0.025* | 0.448 | 0.35 | 0.137 | 0.334 | 0.912 | 0.349 |
| N=28 |  |  |  |  |  |  |  |  |  |
| **IL-13** | 0.241 | 0.737 | 0.178 | 0.896 | 0.151 | 0.157 | 0.377 | 0.028* | 0.151 |
| N=81 |  |  |  |  |  |  |  |  |  |
